# Real-World Evidence of Tolerability of 20% Subcutaneous Immunoglobulin Treatment

**DOI:** 10.1007/s10875-023-01436-4

**Published:** 2023-02-21

**Authors:** Kevin Rosenbach, Michelle Park, Marie Sanchirico, Oliseyenum Nwose, Kenneth Paris

**Affiliations:** 1 Naples Allergy Center, Naples, FL USA; 2 Takeda Development Center Americas, Inc., Cambridge, MA USA; 3 Takeda Pharmaceuticals USA, Inc., Lexington, MA USA; 4 Department of Pediatrics, Louisiana State University Health Sciences Center, Children’s Hospital, New Orleans, LA USA

**Keywords:** Subcutaneous immunoglobulin, Ig20Gly, immunoglobulin replacement therapy, primary immunodeficiency diseases, inborn errors of immunity, real-world usage, real-world experience, chart review

## Abstract

**Purpose:**

The safety and efficacy of subcutaneous immune globulin 20% (human) solution (Ig20Gly) were demonstrated in clinical trials. However, real-world evidence of the tolerability of self-administered Ig20Gly in elderly patients is lacking. We describe real-world patterns of Ig20Gly usage for 12 months in patients with primary immunodeficiency diseases (PIDD) in the USA.

**Methods:**

This retrospective chart review of longitudinal data from 2 centers included patients aged ≥ 2 years with PIDD. Ig20Gly administration parameters, tolerability, and usage patterns were assessed at initial and subsequent 6- and 12-month infusions.

**Results:**

Of 47 enrolled patients, 30 (63.8%) received immunoglobulin replacement therapy (IGRT) within 12 months before starting Ig20Gly, and 17 (36.2%) started IGRT de novo. Patients were predominantly White (89.1%), female (85.1%), and elderly (aged > 65 years, 68.1%; median age = 71.0 years). Most adults received at-home treatment during the study, and most self-administered at 6 months (90.0%) and 12 months (88.2%). Across all time points, infusions were administered at a mean rate of 60–90 mL/h/infusion, using a mean of 2 sites per infusion, on a weekly or biweekly frequency. No emergency department visits occurred, and hospital visits were rare (*n* = 1). Forty-six adverse drug reactions occurred in 36.4% of adults, mostly localized site reactions; none of these or any adverse events led to treatment discontinuation.

**Conclusion:**

These findings demonstrate tolerability and successful self-administration of Ig20Gly in PIDD, including elderly patients and patients starting IGRT de novo.

**Supplementary Information:**

The online version contains supplementary material available at 10.1007/s10875-023-01436-4.

## Introduction

Primary immunodeficiency diseases (PIDD, also referred to as inborn errors of immunity) include over 430 distinct genetic conditions that affect the immune system, including X-linked agammaglobulinemia, common variable immunodeficiency (CVID), and severe combined immunodeficiency [1, 2]. The estimated prevalence of PIDD is 1/1000–1/5000 people, and patients with PIDD often experience chronic, recurrent, and potentially life-threatening infections [3, 4]. To prevent chronic infections, immunoglobulin (IG) replacement therapy (IGRT) is the standard of care for patients with PIDD with defective antibody production [5] and can be administered intravenously (IVIG) or subcutaneously (SCIG). The advantages of SCIG therapy are that it does not require venous access, can be self-administered at home, and is associated with fewer infusion-related systemic adverse drug reactions (ADRs) than IVIG therapy [6]. However, the volume infused per infusion site is lower with SCIG than IVIG, requiring administration via multiple infusion sites typically on a weekly or biweekly basis [7].

Immune globulin subcutaneous (human) 20% solution (Ig20Gly) is an IGRT approved in the USA for use in patients with PIDD aged 2 years and older [8]. Ig20Gly allows for subcutaneous infusion in low volumes, reduces infusion times compared with less concentrated products, and the Ig20Gly treatment regimen can be modified, allowing for treatment individualization [7–9]. The safety and efficacy of Ig20Gly have been demonstrated in 2 pivotal phase 2/3 clinical trials in adult and pediatric patients with PIDD in North America and Europe [7, 9–11]. In these trials, the rates of validated acute serious bacterial infections were 0.01 and 0.02 per patient-year, significantly lower than the threshold of 1.0 event per year specified by the FDA guidelines as providing substantial evidence of efficacy [7, 9]. In a pooled analysis of these trials, no treatment-related serious or severe adverse events (AEs) were reported [10]. However, data regarding the real-world use of Ig20Gly (i.e., patient characteristics during treatment with Ig20Gly, reasons for switching, and administration and infusion parameters, including tolerability of high infusion volumes and rates) in patients with PIDD in the USA are needed, particularly in an elderly population.

To address the need for real-world data, this study retrospectively reviewed medical records of patients with PIDD to gain insights into the real-world usage and administration of Ig20Gly in the USA.

## Methods

### Study Design

**Fig. 1:**
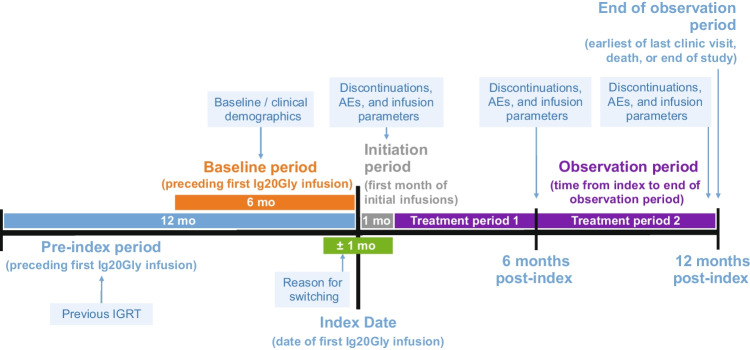
Study design. AE, adverse event; Ig20Gly, immune globulin subcutaneous (human) 20% solution; IGRT, immunoglobulin replacement therapy; mo, months

The medical records of all patients with PIDD who were treated with Ig20Gly at 2 centers in the USA, the National Allergy Center in Naples, FL, and the Louisiana State University Health Science Center, Children’s Hospital in New Orleans, LA, were retrospectively reviewed, and data were collected between December 2019 and March 2021 (Fig. 1). Many patients were on a 3-month follow-up schedule. All patients and their parents or caregivers were informed of the study’s purpose and provided written informed consent. The study was approved by the relevant institutional review boards.

### Patients

The study population consisted of patients diagnosed with PIDD who received Ig20Gly at one of the 2 participating clinical centers in the USA. Patients were eligible for inclusion in this study if they were diagnosed with a form of PIDD involving a defect in antibody formation and requiring IGRT, were ≥ 2 years of age, and had the initial, 6-, and 12-month physician visits. Patients were excluded if they were currently enrolled in another study or trial.

### Data Collection

Observational data at treatment initiation, 6, and 12 months were obtained from patient medical records. Information acquired from the medical records included patient demographics and clinical characteristics, medical history including prior treatment and reasons for selecting or switching to Ig20Gly, infusion parameters of Ig20Gly infusion (e.g., dose, frequency, location of administration, administration setting), tolerability of Ig20Gly, including the incidence of infusion-related ADRs and infections, safety of Ig20Gly, including the incidence of AEs (defined as any medical condition that occurred over the study period), and treatment-emergent AEs (TEAEs; defined as AEs that first occurred or worsened after initiating treatment with Ig20Gly).

The data were additionally assessed by prior IG exposure (patients who were not on IGRT within the prior 12 months [IG-de novo] and patients who switched from another IGRT [IG-experienced]) and by age (pediatric [aged 2 to < 18 years] and adult [aged ≥ 18 years] patients).

### Statistical Analysis

Data on patient characteristics and real-world patterns of Ig20Gly usage and administration were summarized descriptively using means, standard deviations (SDs), medians, and ranges. Continuous variables were expressed in the number of evaluable values, means, SDs, and medians. Categorical values were expressed as frequency counts (absolute and relative). This is a descriptive study; thus, no statistical hypotheses were tested.

## Results

### Patients

Between the 2 participating centers, 67 patients were screened, and 47 patients (44 adults and 3 children) met the eligibility criteria and were enrolled in the study. The patients were predominantly White (89.1%) and not of Hispanic or Latino ethnicity (93.3%). The median (range) age of the three children was 9.0 (8–13) years. All patients (*n* = 47; 100%) were insured, with the majority enrolled in Medicare (*n* = 33; 70.2%).

Among the 44 adult patients, 39 (88.6%) were female, the median (range) age was 71.0 (26–82) years, and 32 (72.7%) were aged > 65 years (Table 1). More than 50% of the adult patients were obese or overweight.

**Table 1:** Patient demographics^a^

	IG-de novo adults	IG-experienced adults	Total adults
Total patients, *n*	16	28	44
Age, median (range), years Age > 65 years, *n* (%)	69.5 (45–82) 10 (62.5)	71.0 (26–81) 22 (78.6)	71.0 (26–82) 32 (72.7)
Sex, *n* (%)			
Female	15 (93.8)	24 (85.7)	39 (88.6)
Male	1 (6.3)	4 (14.3)	5 (11.4)
Race, *n* (%)			
White	14 (87.5)	26 (92.9)	40 (90.9)
Other	2 (12.5)	2 (7.1)	4 (9.1)
Ethnicity, *n* (%)			
Hispanic or Latino	0	2 (7.1)	2 (4.5)
Not Hispanic or Latino	16 (100)	24 (85.7)	40 (90.9)
Body mass index, mean (SD), kg/m^2^	27.5 (7.4)	29.7 (6.9)	28.9 (7.0)

^a^Adult patients; percentages are calculated based on those with available data. Abbreviations: *IG*, immunoglobulin; *IG-de novo*, patients who were not on immunoglobulin replacement therapy within the prior 12 months; *IG-experienced*, patients who were on another immunoglobulin replacement therapy within the prior 12 months and had switched to Ig20Gly; *SD*, standard deviation

Owing to the small number of pediatric patients, Tables 1, 2, 3, and 4 present the results of the adult population, with the available pediatric data summarized in the text.

**Table 2:** Ig20Gly administration at initiation, 6, and 12 months^a^

	IG-de novo adults (*n* = 16)			IG-experienced adults (*n* = 28)		
	Initiation	6 months	12 months	Initiation	6 months^b^	12 months^b^
Infusion setting, *n* (%)						
Home	5 (71.4)	9 (90.0)	8 (100)	10 (76.9)	14 (77.8)	13 (76.5)
Infusion center	2 (28.6)	0	0	1 (7.7)	0	0
Other	0	1 (10.0)	0	2 (15.4)	4 (22.2)	4 (23.5)
Infusion administrator, *n* (%)						
Self-administered	0	8 (88.9)	7 (87.5)	0	10 (90.9)	8 (88.9)
Received assistance	7 (100)	1 (11.1)	1 (12.5)	12 (100)	1 (9.1)	1 (11.1)
Anatomical location, *n* (%)						
Thigh	4 (57.1)	4 (100)	6 (100)	15 (75.0)	0	0
Abdomen	3 (42.9)	0	0	4 (20.0)	0	0
Arm	0	0	0	1 (5.0)	0	0

^a^Adult patients; percentages are calculated based on those with available data. ^b^Anatomical location was not reported for any patients. Abbreviations: *IG*, immunoglobulin; *Ig20Gly*, immune globulin subcutaneous (human) 20% solution; *IG-de novo*, patients who were not on immunoglobulin replacement therapy within the prior 12 months; *IG-experienced*, patients who were on another immunoglobulin replacement therapy within the prior 12 months and switched to Ig20Gly

**Table 3:** Ig20Gly infusion parameters at treatment initiation, 6, and 12 months^a^

	IG-de novo (*n* = 16)			IG-experienced (*n* = 28)		
	Initiation	6 months	12 months	Initiation	6 months	12 months
Dose, *n*	9	16	16	19	28	28
Mean (SD), g	8.2 (1.2)	9.4 (2.7)	10.3 (3.7)	15.9 (5.6)	15.3 (5.8)	16.5 (5.6)
Infusion rate, *n*	7	8	9	12	6	6
Mean (SD), mL/h ≥ 120 mL/h, *n*	55.3 (21.1) 0	66.0 (34.5) 2	79.0 (33.7) 1	94.0 (20.5) 2	63.0 (32.0) 1	68.0 (15.5) 0
Sites per infusion, *n*	7	2	3	12	0	0
Mean (SD), sites/infusion	1.6 (0.5)	2.0 (0)	2.0 (0)	1.8 (0.4)	n/a	n/a
Infusion duration, *n*	7	8	9	12	6	6
Mean (SD), min	49.3 (16.7)	44.4 (17.6)	44.4 (14.2)	51.2 (17.9)	59.2 (14.6)	69.2 (22.9)
Dose adjustments, *n*						
Due to a local AE	0	1	0	0	0	0
Due to a systemic AE	0	0	0	0	0	0
No reason noted	0	2	0	0	0	1
Other reason	0	1	3	0	0	2
Not reported	7	0	0	10	1	1

^a^Adult patients with available data. Abbreviations: *AE*, adverse event; *IG*, immunoglobulin; *Ig20Gly*, immune globulin subcutaneous (human) 20% solution; *IG-de novo*, patients who were not on immunoglobulin replacement therapy within the prior 12 months; *IG-experienced*, patients who were on another immunoglobulin replacement therapy and switched to Ig20Gly within the prior 12 months; *SD*, standard deviation

**Table 4:** Adverse drug reactions^a^

Adverse reaction type, *n* (%) events	IG-de novo (*n* = 16)	IG-experienced (*n* = 28)	Total (*n* = 44)
Patients with at least 1 ADR	7 (43.8) 20	9 (32.1) 26	16 (36.4) 46
General disorders and administration site conditions	7 (43.8) 19	8 (28.6) 25	15 (34.1) 44
Infusion site pain	4 (25.0) 4	4 (14.3) 14	8 (18.2) 18
Infusion site erythema	2 (12.5) 3	1 (3.6) 4	3 (6.8) 7
Infusion site pruritus	2 (12.5) 5	1 (3.6) 1	3 (6.8) 6
Infusion site reaction	2 (12.5) 2	1 (3.6) 1	3 (6.8) 3
Fatigue	1 (6.3) 1	1 (3.6) 1	2 (4.5) 2
Infusion site extravasation	1 (6.3) 1	1 (3.6) 1	2 (4.5) 2
Infusion site rash	1 (6.3) 1	1 (3.6) 1	2 (4.5) 2
Infusion site swelling	0	2 (7.1) 2	2 (4.5) 2
Infusion site hemorrhage	1 (6.3) 1	0	1 (2.3) 1
Infusion site warmth	1 (6.3) 1	0	1 (2.3) 1
Musculoskeletal and connective tissue disorders	0	1 (3.6) 1	1 (2.3) 1
Myalgia	0	1 (3.6) 1	1 (2.3) 1
Nervous system disorders	1 (6.3) 1	0	1 (2.3) 1
Headache	1 (6.3) 1	0	1 (2.3) 1

^a^Adult patients with available data. Abbreviations: *ADR*, adverse drug reaction; *IG*, immunoglobulin; *Ig20Gly*, immune globulin subcutaneous (human) 20% solution; *IG-de novo*, patients who were not on immunoglobulin replacement therapy within the prior 12 months; *IG-experienced*, patients who were on another immunoglobulin replacement therapy within the prior 12 months and switched to Ig20Gly

### Medical History

Most (*n* = 30; 63.8%) patients had received an IGRT within the 12-month period before starting Ig20Gly treatment, and the remaining patients (*n* = 17; 36.2%) started de novo on Ig20Gly. Prior IGRTs included 10% IVIG (*n* = 31), facilitated 10% SCIG with recombinant human hyaluronidase (*n* = 16), 20% SCIG (*n* = 9), and unspecified IVIG (*n* = 1). Reasons provided for switching to Ig20Gly included insurance (*n* = 6; 20.0%), an unfavorable AE profile with previous treatment (*n* = 6; 20.0%), preference for less frequent infusions/less travel to an infusion center (*n* = 4; 13.3%), previous treatment was not effective (*n* = 2; 6.7%), higher infusion volumes, and preference for at-home use (*n* = 1; 3.3% each). The reason for switching was not indicated for 8 (26.7%) patients and was listed as “other” for 2 (6.7%) patients.

Most (97.9%) patients had received at least 1 concomitant medication, with 43 (91.5%) patients had received more than 5 medications. The 5 most common classes of concomitant medications patients had received were inhalant adrenergic (61.7%), drugs for peptic ulcer and gastroesophageal reflux disease (57.4%), antihistamines (systemic; 55.3%), cardiac stimulants excluding glycosides (55.3%), and direct-acting antivirals (48.9%). The concomitant use of antithrombotic agents was low (19.1%). All patients had at least 1 comorbidity, which was the most frequent history of infections and infestations (*n* = 44; 93.6%), followed by respiratory, thoracic, and mediastinal disorders (*n* = 41; 87.2%), gastrointestinal disorders (*n* = 38; 80.9%), musculoskeletal and connective tissue disorders (*n* = 38; 80.9%), and immune system disorders (*n* = 34; 72.3%). Few patients had a history of deep vein thrombosis (8.5%) and pulmonary embolism (6.4%).

The most common PIDD diagnosis was CVID (74.5%), followed by selective polysaccharide antibody deficiency (12.8%), humoral immune defect (4.3%), ataxia telangiectasia (2.1%), combined immunodeficiency (2.1%), Good syndrome (2.1%), and selective IgG subclass deficiency (2.1%). Median (range) disease duration was 187.0 (5–4717) days and was numerically shorter in IG-de novo patients than in IG-experienced patients (24.0 [5–480] and 805.0 [8–4717] days, respectively). Patient age (median [range]) was similar at PIDD diagnosis for IG-de novo (66.0 [9.0–80.0] years) and IG-experienced patients (66.0 [0.5–77.0] years). The most reported PIDD-related symptom was frequent and recurrent infections (45 [95.7%] patients).

#### Ig20Gly Administration

Table 2 reports Ig20Gly administration parameters at initiation, 6, and 12 months. Most adult patients received treatment at home across all time points (75.0%, 82.1%, and 84.0% at initiation, 6, and 12 months, respectively). The proportion of adult IG-de novo patients who received treatment at home increased over time (71.4%, 90.0%, and 100.0% at initiation, 6, and 12 months, respectively), while the proportion of adult IG-experienced patients who received treatment at home remained consistent throughout the study (76.9%, 77.8%, and 76.5% at initiation, 6, and 12 months, respectively). All adult patients with available data received assistance with their initiation infusion, and most self-administered Ig20Gly at 6 months (90.0%) and 12 months (88.2%). Similar proportions of adult IG-de novo and IG-experienced patients self-administered Ig20Gly at 6 months (88.9% vs 90.9%, respectively) and 12 months (87.5% vs 88.9%, respectively). The thigh was the most common infusion site for adult patients with available data at all time points (70.4%, 100.0%, and 100.0% at initiation, 6, and 12 months, respectively).

Of pediatric patients (*n* = 3) with available data, all received their initial infusion at home with nursing assistance. At 6 and 12 months, all 3 pediatric patients infused Ig20Gly at home with assistance from their adult caregiver. Two pediatric patients with available data were reported as having the abdomen as the infusion site.

#### Infusion Parameters

##### Overall Adults

IG dose (mean [SD]) for adult patients was similar over time, with a slightly higher dose at 12 months (13.4 [5.9], 13.1 [5.6], and 14.2 [5.8] g at initiation, 6, and 12 months, respectively). The mean (SD) infusion rate was 79.7 (27.8), 64.7 (32.2), and 74.6 (27.7) mL/h per infusion at initiation, 6, and 12 months, respectively. Several patients (including 2 patients at initiation, 3 patients at 6 months, and 1 patient at 12 months) received treatment at a rate ≥ 120 mL/h, using 2 infusion sites (Table 3). The mean (SD) number of infusion sites per infusion at Ig20Gly initiation was 1.7 (0.5). Infusion duration (mean [SD]) was similar across time, with a slightly higher duration at 12 months (50.5 [17.0], 50.7 [17.5], and 54.3 [21.5] min at initiation, 6, and 12 months, respectively). The prescribed infusion frequency for adults with available data was weekly or biweekly at initiation (57.1% vs 42.9%), 6 months (50.0% vs 50.0%), and 12 months (44.2% vs 55.8%). The need for dose adjustments among adult patients was minimal (10 instances) within the 12-month study duration (Table 3).

##### IG-De Novo Adults

In the adults who started IGRT de novo, the mean (SD) IgG level was 714.5 (141.3) mg/dL at Ig20Gly initiation. IG dose (mean [SD]) increased from initiation (8.2 [1.2] g) to 12 months (10.3 [3.7] g; Table 3). The infusion rate (mean [SD]) also increased from initiation (55.3 [21.1] mL/h) to 12 months (79.0 [33.7] mL/h; Table 3). The mean (SD) number of infusion sites per infusion was approximately 2 throughout the study period (Table 3). The infusion duration (mean [SD]) slightly decreased over time (49.3 [16.7] and 44.4 [14.2] min at initiation and 12 months, respectively; Table 3).

##### IG-Experienced Adults

The mean (SD) IgG level in the adults who switched from another IGRT was 719.6 (309.9) mg/dL, similar to IG-de novo adults. IG dose (mean [SD]) was similar across time, with a slightly higher dose at 12 months (15.9 [5.6], 15.3 [5.8], and 16.5 [5.6] g at initiation, 6, and 12 months, respectively). At Ig20Gly initiation, adult IG-experienced patients used a mean (SD) of 1.8 (0.4) infusion sites per infusion (Table 3). Unlike the IG-de novo adults, infusion rate (mean [SD]) decreased over time (94.0 [20.5] and 68.0 [15.5] mL/h at initiation and 12 months, respectively), and infusion duration (mean [SD]) increased (51.2 [17.9] and 69.2 [22.9] min at initiation and 12 months, respectively; Table 3).

##### Pediatric Patients (*n* = 3)

The mean (SD) IG dose administered in children was 5.7 (1.5), 6.3 (2.1), and 6.3 (2.1) g at initiation, 6, and 12 months, respectively. For pediatric patients with available data at initiation (*n* = 2), the mean (SD) infusion rate was 60.0 (6) mL/h (no pediatric data were available at 6 months), and the mean (SD) infusion duration at initiation was 30.0 (0) min (no pediatric data were available at 6 or 12 months). The prescribed infusion frequency was weekly or biweekly at initiation (33.3% vs 66.7%), 6 months (50.0% vs 50.0%), and 12 months (33.3% vs 66.7%). No pediatric patients required dose adjustments during the study period. Overall, 1 child required additional therapy due to hospital formulary limitations during hospitalization (gammagard liquid 10%).

### Healthcare Resource Utilization

There were no emergency department visits during the study. One adult patient had 3 hospital visits (due to pneumonia, flu, and interstitial lung disease); each visit had a duration of less than 1 day. Twenty-one adult patients (IG-de novo, *n* = 9; IG-experienced, *n* = 12) had a median (range) of 4.0 (1–5) physician visits, with many patients on a 3-month follow-up schedule. Infections were reported in 6 adult patients during the 12-month study period. One pediatric patient was admitted to the hospital for an issue unrelated to the study, and no pediatric patients had physician visits.

### Infections

Six patients (all adults) had a mean (SD) of 2.3 (1.8) infections. Three (6.4%) patients each had 1 infection, and 1 (2.1%) patient each had 2, 4, or 5 infections.

### Tolerability

A total of 46 ADRs occurred in 16 (36.4%) adult patients (Table 4). ADRs were most frequently categorized as general disorders and administration site conditions (44 events in 15 adult patients). The most common (> 2 events) ADRs were infusion site pain, infusion site erythema, infusion site pruritus, and infusion site reaction. None of the ADRs were associated with treatment discontinuation or a change in dose. Three ADRs resulted in further action: 1 event (infusion site extravasation) was followed by a switch in needle size; 1 event (infusion site rash) was followed by a change of infusion site from the leg to the abdomen; and 1 patient received concomitant medication for an infusion site rash.

Among pediatric patients (*n* = 3), 5 ADRs occurred in 1 (33.3%) patient, including infusion site pain (1 event in 1 patient) and infusion site erythema (4 events in 1 patient), none of which were associated with treatment discontinuation or a change in dose.

### Safety

A total of 336 AEs (defined as any medical condition that occurred over the study period) were reported in 47 (100.0%) patients, with the majority considered not related to PIDD (1 event in 1 [2.1%] patient was considered to be associated with PIDD). Concomitant medication was required for 37 (78.7%) patients who experienced AEs, and none of the AEs led to Ig20Gly discontinuation. There were 21 serious AEs (SAEs) reported in 13 (27.7%) patients: none led to treatment discontinuation and were not considered associated with PIDD; 12 required concomitant medication in 10 (21.3%) patients. A total of 105 TEAEs were experienced by 44.7% of patients, with system organ classes reported in > 10% of patients that were general disorders and administration site conditions (49 events in 13 [27.7%] patients) and infections and infestations (10 events in 8 [17.0%] patients).

## Discussion

This study aimed to describe real-world patterns of Ig20Gly usage and administration in patients with PIDD in the USA. The findings provide further evidence that Ig20Gly can be successfully administered by patients in a home setting with minimal need for dose adjustments and a low incidence of ADRs. Most patients were able to self-administer Ig20Gly throughout the 12 months of the study after assistance with the first administration. Notably, more than 70% of patients received treatment at home across all time points, similar to percentages of home administration reported in the pivotal trials of Ig20Gly in North America (79.1%) and Europe (74.1%) [7, 9]. This study population included patients initiating Ig20Gly de novo (36.2%) and most patients over 65 years of age (68.1%), supporting the potential for these particular patient groups to benefit from self-administered infusions at home.

The administered dose of Ig20Gly was stable throughout the study period, with a slightly higher dose (14 g) at 12 months. These median doses were within the range of the median (interquartile range) dose received at the fourth infusion (12 [10–20] g) in a real-world population of patients with PIDD [12]. The mean infusion rates achieved during the present study (approximately 60–90 mL/h) were similar to the median maximum infusion rate in the pivotal North American clinical trial (60 mL/h) [9] and were greater than the median maximum infusion rate reported in the European clinical trial (20 mL/h) [7]. The mean infusion rate decreased over time in IG-experience adults; however, the observation of trends is limited by the low number of patients with available data.

The mean infusion duration remained approximately 1 h across all time points, comparable to the median infusion duration observed in the pivotal trials (0.95 h for both) [7, 9] and the real-world study of Ig20Gly usage (50 min) [12]. Patients received treatment weekly or every 2 weeks, with a greater proportion receiving biweekly infusions by 12 months, which aligns with the infusion frequencies in the pivotal trials (from daily up to biweekly) [7–9]. Furthermore, the need for dose adjustments or additional therapy was minimal over the course of the 12-month period.

Most patients had received IGRT within the 12-month period before starting Ig20Gly treatment, citing insurance issues and an unfavorable AE profile with previous treatment as the most common reasons for switching to Ig20Gly. For the 17 (36.2%) patients who had not received IG treatment in the 12 months before the study, dose adjustments or treatment ramp-up might be expected to occur more frequently. The mean dose slightly increased over time in adult patients, and adult IG-de novo patients received a consistently lower dose (from 8.2 g at initiation to 10.3 g at 12 months) than the IG-experienced patients who had switched from a recent IGRT (from 15.9 g at initiation to 16.5 g at 12 months). These findings are in line with what is typically seen in real-world practice, where dosing tends to increase rather than decrease with longer IGRT experience (e.g., due to rounding according to the nearest full vial size) [12]. At least 4 patients requested and tolerated faster infusion rates (approximately 24 g over 1 h) over time.

The tolerability of Ig20Gly was consistent with results from previous clinical studies, and no patients discontinued Ig20Gly for any reason [9–11]. The incidence of ADRs was low, and none of the reported ADRs led to treatment discontinuation, demonstrating the real-world tolerability findings in this study were consistent with clinical trials [7, 9]. The most common local ADRs (infusion site pain, infusion site erythema, infusion site pruritus, and infusion site reaction) were consistent with the clinical trials [7, 9]. Similar ADRs were also reported in a study of home-based SCIG therapy; therefore, this is the second study to demonstrate successful self-administration of SCIG in elderly patients with PIDD [13]. After initial SCIG infusions, the incidence of local site reactions decreased in real-world studies that included IG-naïve patients [14, 15]. IG-de novo patients in this study tolerated Ig20Gly, adding to previous evidence that IG-naïve patients are safely and effectively treated when initiating SCIG [16, 17]. Similarly, initiation of SCIG was well-tolerated and effective in a study of elderly IG-naïve patients in which mild or moderate local site reactions did not result in treatment discontinuation [18].

The data collected on comorbid medical conditions that occurred over the study period (defined as AEs in this study) included many events that would be expected medical conditions or symptoms in the patients in this study who were mostly of advanced age and covered by Medicare. Most of these AEs were not considered related to PIDD, and none led to death or treatment discontinuation.

In addition, healthcare utilization was low during the 12-month treatment period, with no emergency department visits and 1 adult patient with hospitalization. During the 12-month treatment period, 21 adult patients had a median of 4 physician visits, and infections were reported in 6 adult patients. These results correspond with the low hospitalizations consistently reported with SCIG therapy [6, 9, 19].

Several limitations require consideration when interpreting the findings of this retrospective observational study. All parameters were limited to the availability and accuracy of the data source and collection procedures across the clinical centers. The data collected were dependent on the accuracy and extent of completeness of reporting by patients receiving care at home. Thus, information on tolerability may not have been fully captured because most infusions were performed outside of the clinical setting. Furthermore, missing data may confound the trends that were observed in the administration and infusion parameters over time.

There are various factors to consider regarding the generalizability of these findings. Data for the IG-de novo (*n* = 17) and pediatric (*n* = 3) subgroups should be generalized with caution given the low number of patients per group. While the majority of adults in the IG-de novo subgroup were over 65 years of age, the small sample size (*n* = 10) should be considered when interpreting the data for elderly patients starting Ig20Gly de novo. In addition, because it is not known whether IG-de novo patients received IGRT before the 12-month pre-index period, these patients may not be representative of those who have never received IGRT. It should also be noted that due to the low number of patients in each group, the results could not be further stratified by the type of prior IGRT in the IG-experienced patients (i.e., IVIG, 10% SCIG, 20% SCIG, or facilitated SCIG). Selection bias may have been introduced by limiting study enrollment to patients with a follow-up period of ≥ 12 months. Furthermore, patients selected for this study were identified from specific clinical centers and may not represent the overall population of patients with PIDD initiating Ig20Gly treatment in the USA. Given the most common health insurance provider (i.e., Medicare) and the advanced age of the majority of the patients in this study, these findings may be most representative of a Medicare-eligible US population of older adults rather than commercially insured and younger populations. However, our results add to the observation that elderly patients can be successfully treated with SCIG in the home setting. In a separate study of elderly patients with PIDD, most patients self-administered weekly SCIG infusions, and the mean infusion duration was 65.3 min [13].

The results of this retrospective medical record review study improve understanding of real-world Ig20Gly usage in patients with PIDD in the USA. Most patients in this study successfully self-administered Ig20Gly in a home setting with minimal need for additional therapy or adjustments to dose or infusion rate. The tolerability findings support data reported in clinical trials, as evidenced by no emergency department visits, low hospitalization rates, and no ADRs resulting in treatment discontinuation. This study provides real-world evidence confirming the effectiveness and tolerability of Ig20Gly in an elderly, at-risk population.

## Supplementary Information

Supplementary Material Supplemental Table S1. Ig20Gly Infusion Parameters at Treatment Initiation, 6, and 12 Months – by Adult Age Group Supplemental Table S2. Adverse Drug Reactions – by Adult Age Group (DOCX 57 kb)

## Data Availability

The data sets generated and/or analyzed during the current study are available from the corresponding author on reasonable request to researchers who provide a methodologically sound proposal. The data will be provided after their de-identification, in compliance with data protection anonymization.
