# Supplementary material for: Real-World Evidence of Tolerability of 20% Subcutaneous Immunoglobulin Treatment
Source: J Clin Immunol. 2023 Feb 21;43(5):912–20. doi: 10.1007/s10875-023-01436-4 (PMC10275800; doi:10.1007/s10875-023-01436-4)
Supplement: Supplementary file 1 — Supplemental Table S1. Ig20Gly Infusion Parameters at Treatment Initiation, 6, and 12 Months – by Adult Age Group Supplemental Table S2. Adverse Drug Reactions – by Adult Age Group (DOCX 57 kb) [file 10875_2023_1436_MOESM1_ESM.docx]

**Supplemental Materials**

**Supplemental Table S1. Ig20Gly Infusion Parameters at Treatment Initiation, 6, and 12 Months – by Adult Age Group**

|  | **IG-de novo**  **(n = 16)** | | | | | | **IG-experienced**  **(n = 28)** | | | | | |
| --- | --- | --- | --- | --- | --- | --- | --- | --- | --- | --- | --- | --- |
|  | **Age < 65 years**  **(n = 6)** | | | **Age ≥ 65 years**  **(n = 10)** | | | **Age < 65 years**  **(n = 6)** | | | **Age ≥ 65 years**  **(n = 22)** | | |
|  | **Initiation** | **6 Months** | **12 Months** | **Initiation** | **6 Months** | **12 Months** | **Initiation** | **6 Months** | **12 Months** | **Initiation** | **6 Months** | **12 Months** |
| Dose, n | 2 | 6 | 6 | 7 | 10 | 10 | 3 | 6 | 6 | 16 | 22 | 22 |
| Mean (SD), g | 9.0 (1.4) | 9.2 (2.7) | 9.3 (2.4) | 8.0 (1.2) | 9.6 (2.8) | 10.8 (4.3) | 20.7 (5.8) | 20.3 (3.7) | 20.3 (3.7) | 15.0 (5.3) | 13.9 (5.5) | 15.4 (5.6) |
| Infusion rate, n | 2 | 3 | 2 | 5 | 5 | 7 | 1 | 1 | 2 | 11 | 5 | 4 |
| Mean (SD), mL/h  ≥ 120 mL/h, n | 51.0 (4.2)  0 | 75.0 (39.0)  1 | 78.0 (25.5)  0 | 57.0 (25.5)  0 | 60.6 (35.0)  1 | 79.3 (37.5)  1 | 81.0 (n/a)  0 | 120.0 (n/a)  1 | 66.0 (0)  0 | 95.2 (21.1)  2 | 51.6 (17.5)  0 | 69.0 (19.9)  0 |
| Sites per infusion, n | 2 | 1 | 1 | 5 | 1 | 2 | 1 | 0 | 0 | 11 | 0 | 0 |
| Mean (SD), sites/infusion | 1.0 (0) | 2.0 (n/a) | 2.0 (n/a) | 1.8 (0.4) | 2.0 (n/a) | 2.0 (0) | 2.0 (n/a) | n/a | n/a | 1.8 (0.4) | n/a | n/a |
| Infusion duration, n | 2 | 3 | 2 | 5 | 5 | 7 | 1 | 1 | 2 | 11 | 5 | 4 |
| Mean (SD), min | 52.5 (3.5) | 31.7 (12.6) | 42.5 (24.7) | 48.0 (20.2) | 52.0 (16.4) | 45.0 (12.9) | 90.0 (n/a) | 60.0 (n/a) | 90.0 (2.0) | 47.6 (13.7) | 59.0 (16.4) | 58.8 (21.0) |
| Dose adjustments, n |  |  |  |  |  |  |  |  |  |  |  |  |
| Due to a local AE | 0 | 0 | 0 | 0 | 1 | 0 | 0 | 0 | 0 | 0 | 0 | 0 |
| Due to a systemic AE | 0 | 0 | 0 | 0 | 0 | 0 | 0 | 0 | 0 | 0 | 0 | 0 |
| No reason noted | 0 | 0 | 0 | 0 | 2 | 0 | 0 | 0 | 0 | 0 | 0 | 1 |
| Other reason | 0 | 0 | 0 | 0 | 1 | 3 | 0 | 0 | 0 | 0 | 0 | 2 |
| Not reported | 4 | 0 | 0 | 3 | 0 | 0 | 4 | 0 | 0 | 6 | 1 | 1 |

^a^Adult patients with available data.

Abbreviations: AE, adverse event; IG, immunoglobulin; Ig20Gly, immune globulin subcutaneous (human) 20% solution; IG-de novo, patients who were not on immunoglobulin replacement therapy within the prior 12 months; IG-experienced, patients who were on another immunoglobulin replacement therapy and switched to Ig20Gly within the prior 12 months; SD, standard deviation.

**Supplemental Table S2. Adverse Drug Reactions – by Adult Age Group**

| **Adverse Reaction Type, n (%) Events** | **IG-de novo**  **(n = 16)** | | **IG-experienced**  **(n = 28)** | | **Total**  **(N = 44)** |
| --- | --- | --- | --- | --- | --- |
|  | **Age < 65 years**  **(n = 6)** | **Age ≥ 65 years**  **(n = 10)** | **Age < 65 years**  **(n = 6)** | **Age ≥ 65 years**  **(n = 22)** |  |
| Patients with at least 1 ADR | 3 (50.0) 13 | 4 (40.0) 7 | 2 (33.3) 12 | 7 (31.8) 14 | 16 (36.4) 46 |
| General disorders and administration site conditions | 3 (50.0) 13 | 4 (40.0) 6 | 2 (33.3) 12 | 6 (27.3) 13 | 15 (34.1) 44 |
| Infusion site pain | 3 (50.0) 3 | 1 (10.0) 1 | 1 (16.7) 5 | 3 (13.6) 9 | 8 (18.2) 18 |
| Infusion site erythema | 1 (16.7) 2 | 1 (10.0) 1 | 1 (16.7) 4 | 0 | 3 ( 6.8) 7 |
| Infusion site pruritus | 2 (33.3) 5 | 0 | 0 | 1 (4.5) 1 | 3 ( 6.8) 6 |
| Infusion site reaction | 0 | 2 (20.0) 2 | 0 | 1 (4.5) 1 | 3 ( 6.8) 3 |
| Fatigue | 1 (16.7) 1 | 0 | 1 (16.7) 1 | 0 | 2 ( 4.5) 2 |
| Infusion site extravasation | 1 (16.7) 1 | 0 | 1 (16.7) 1 | 0 | 2 ( 4.5) 2 |
| Infusion site rash | 0 | 1 (10.0) 1 | 0 | 1 (4.5) 1 | 2 ( 4.5) 2 |
| Infusion site swelling | 0 | 0 | 1 (16.7) 1 | 1 (4.5) 1 | 2 ( 4.5) 2 |
| Infusion site hemorrhage | 1 (16.7) 1 | 0 | 0 | 0 | 1 ( 2.3) 1 |
| Infusion site warmth | 0 | 1 (10.0) 1 | 0 | 0 | 1 ( 2.3) 1 |
| Musculoskeletal and connective tissue disorders | 0 | 0 | 0 | 1 (4.5) 1 | 1 ( 2.3) 1 |
| Myalgia | 0 | 0 | 0 | 1 (4.5) 1 | 1 ( 2.3) 1 |
| Nervous system disorders | 0 | 1 (10.0) 1 | 0 | 0 | 1 ( 2.3) 1 |
| Headache | 0 | 1 (10.0) 1 | 0 | 0 | 1 ( 2.3) 1 |

^a^Adult patients with available data.

Abbreviations: ADR, adverse drug reaction; IG, immunoglobulin; Ig20Gly, immune globulin subcutaneous (human) 20% solution; IG-de novo, patients who were not on immunoglobulin replacement therapy within the prior 12 months; IG-experienced, patients who were on another immunoglobulin replacement therapy within the prior 12 months and switched to Ig20Gly.
